# Arc-Jet Tests of Carbon–Phenolic-Based Ablative Materials for Spacecraft Heat Shield Applications

**DOI:** 10.3390/ma16103717

**Published:** 2023-05-13

**Authors:** Rajesh Kumar Chinnaraj, Young Chan Kim, Seong Man Choi

**Affiliations:** Department of Aerospace Engineering, Jeonbuk National University, Jeonju 54896, Republic of Korea; rajkumchin@jbnu.ac.kr (R.K.C.); kyc0420@jbnu.ac.kr (Y.C.K.)

**Keywords:** TPS, spacecraft heat shield, ablative materials, plasma wind tunnel, carbon–phenolic, silica–phenolic, atmospheric re-entry

## Abstract

We developed and tested two carbon–phenolic-based ablators for future Korean spacecraft heat shield applications. The ablators are developed with two layers: an outer recession layer, fabricated from carbon–phenolic material, and an inner insulating layer, fabricated either from cork or silica–phenolic material. The ablator specimens were tested in a 0.4 MW supersonic arc-jet plasma wind tunnel at heat flux conditions ranging from 6.25 MW/m^2^ to 9.4 MW/m^2^, with either specimen being stationary or transient. Stationary tests were conducted for 50 s each as a preliminary investigation, and the transient tests were conducted for ~110 s each to stimulate a spacecraft’s atmospheric re-entry heat flux trajectory. During the tests, each specimen’s internal temperatures were measured at three locations: 25 mm, 35 mm, and 45 mm from the specimen stagnation point. During the stationary tests, a two-color pyrometer was used to measure specimen stagnation-point temperatures. During the preliminary stationary tests, the silica–phenolic-insulated specimen’s reaction was normal compared to the cork-insulated specimen; hence, only the silica–phenolic-insulated specimens were further subjected to the transient tests. During the transient tests, the silica–phenolic-insulated specimens were stable, and the internal temperatures were lower than 450 K (~180 °C), achieving the main objective of this study.

## 1. Introduction

The carbon–phenolic-based ablative materials (CPBAMs) are widely used heat shield (thermal protection system—TPS) materials for interplanetary earth return (re-entry) space missions. The examples of CPBAMs used for space missions are AVCOAT (Apollo) [1], PICA (Stardust) [2], and MC-CFRP (Hayabusa) [3]. The use of CPBAMs has become the current industrial norm for spacecraft heat shields; for instance, AVCOAT has been employed again for the Artemis moon mission program’s Orion spacecraft [4], and PICA-X, a variant of PICA used for SpaceX’s Dragon class spacecraft [5]. Apart from spacecraft heat shield applications, CPBAMs are also used for solid propellant rocket motor nozzles, including those used for US space shuttle missions [6] and surface-to-air missiles [7]. Carbon–phenolic materials are typically made by impregnating carbon fibers or felts with a phenolic resin. The thermal degradation or pyrolysis of phenolic resin is an endothermic process that helps to absorb a fraction of heat generated during atmospheric re-entry. The gases produced during the pyrolysis process percolate to the exposed surface and cause a blockage effect, further decreasing the amount of heat transferred to the ablating surface. Hence, CPBAMs are more favored than other types of TPS materials. More details about the carbon–phenolic pyrolysis mechanism can be found in [8], and a broader review of ablative materials for various thermal protection applications can be found in [9], along with various material testing techniques and a list of widely used ablation simulation codes. Carbon–phenolic materials belong to the category of non-reusable charring TPS materials and are known to produce a residual char layer during the pyrolysis process. The thermal properties of a carbon–phenolic material depend on its constituent materials and their structural arrangement, which can be tailored according to the specific application’s requirements. Therefore, understanding the various thermal properties of carbon–phenolic materials is crucial. However, many of the current techniques for measuring the thermal properties of carbon–phenolic materials are old, and new techniques are necessary [10]. Carbon–phenolic materials are not good thermal insulators but act as excellent ablators [11]. The poor thermal insulation, which is characterized by high thermal conductivity, makes carbon–phenolic materials ideal for improving heat dissipation in electronic components [12]. On the contrary, for the spacecraft heat shield applications, the thermal insulation provided by carbon–phenolic materials needs to be improved to protect the metallic frame of the spacecraft, as the commonly used high-temperature resistant aluminum alloys begin to lose their strength above 473.15 K (i.e., 200 °C) [13]. Therefore, from a spacecraft heat shield design perspective, it is important to maintain the back face temperature of the heat shield below 473.15 K (i.e., 200 °C) both during the atmospheric re-entry phase and the after-landing cool-off phase. Insufficient heat shield material thermal insulation properties will result in a thicker heat shield, thereby increasing the overall weight of the spacecraft. For the future Korean spacecraft application, a conservative value of 453.15 K (i.e., 180 °C) is set as the maximum heat shield back face temperature, i.e., as a design limit.

In a heat shield with CPBAMs, the overall thermal insulation properties can be improved through two methods: (1) by reducing the density of the carbon–phenolic material by altering its microstructures and composition, which reduces thermal conductivity but also weakens the material [14], or (2) by adding an inner insulating layer with a lower thermal conductivity to the outer carbon–phenolic recession layer [15].

In our previous study [16], we conducted a preliminary investigation of carbon–phenolic materials with two lamination angles (0° and 30°) and two specially designed SiC-coated carbon–carbon composite specimens (with either cork or graphite base) using an HVOF (high-velocity oxygen fuel) material ablation test facility. In that previous study, the heat flux test conditions were selected to correspond with a re-entry heat flux trajectory of an interplanetary sample return spacecraft, and the values ranged from 3.25 to 11.5 MW/m^2^. After evaluating overall test results, we have selected the carbon–phenolic material with a 30° lamination angle for further development for future Korean spacecraft heat shield applications; however, during the tests, the material’s internal temperatures rose higher than the design limit of 453.15 K (i.e., 180 °C), indicating the need for further improvement. In the same study, it was observed that the internal temperature values of the SiC-coated carbon–carbon composite specimen with a cork material base trended slightly above or closer to the design limit of 453.15 K (i.e., 180 °C). It was also observed that, for that specimen, the cork material section was charred and separated from the SiC-coated section at the carbon adhesive layer, which was used to bond the SiC-coated section with the cork material section.

Based on the results and observations from our previous study, for the current study, we fabricated CPBAM specimens using the carbon–phenolic material with a 30° lamination angle, which we developed in the previous study, as the outer recession layer, and the cork material as the inner insulation layer, to explore the possibility of maintaining the internal temperatures within the design limit of 453.15 K (i.e., 180 °C). We also fabricated another set of CPBAM specimens, in which we used silica–phenolic as the insulation layer instead of the cork material. The outer recession layers and inner insulation layers were bonded together mechanically to ensure structural integrity instead of using the carbon adhesive which was used in the previous study. For more details on specimen fabrication, see Section 2.

The ablation mechanism of silica–phenolic materials is also extensively studied for high-temperature TPS applications [17,18,19,20,21], and two known examples of silica–phenolic ablators used for spacecraft heat shields are Aleastrasil [22] for the European Space Agency (ESA)’s Atmospheric Reentry Demonstrator (ARD) and AQ60/I for the ESA’s Huygens mission to Titan, a moon of Saturn [23]. 

Cork is known for its low density, high compressibility, resilience to vibration, excellent stability, and low thermal conductivity. These combinations of properties have made cork and cork-based materials very useful for various TPS applications. The low thermal conductivity of cork is attributed to the air trapped within its structure [24]. Cork materials have been a part of space technology since the beginning, and their merits have been well-proven. A limitation of cork materials is their ability to withstand only low heat flux conditions. As a result, cork materials are traditionally used for spacecraft back or aft covers or in locations that are not directly exposed to atmospheric re-entry flow. However, cork materials can be used for spacecraft forward heat shields in Mars entry missions or other similar missions where the entry heat fluxes are typically very low. For example, the ESA’s Beagle 2 mission to Mars used a cork phenolic material for its front heat shield, where the estimated Mars entry heat flux was 0.72 MW/m^2^ [2]. Apart from spacecraft applications, the usage of cork materials includes sounding rockets [25] and launch vehicles [26].

The investigations of the specimens were carried out by exposing the specimens to flows generated using the JBNU’s 0.4 MW supersonic arc-jet plasma wind tunnel (PWT). The purpose of this ground testing facility is to simulate atmospheric re-entry conditions by producing high enthalpy and supersonic air plasma. This facility is one of only two academic PWTs in Korea, the other being a 2.4 MW supersonic arc-jet PWT, which also belongs to JBNU. A comparison of various global PWTs capable of simulating atmospheric re-entry conditions can be found in [27].

The specimens that were tested were of two different sizes. A smaller size was used for the preliminary investigation at a stationary test condition, while a larger size was used for a transient test condition selected to stimulate a spacecraft’s re-entry heat flux trajectory.

Most studies [5,14,15,28,29,30,31,32,33,34,35] have only included stationary test conditions, in which specimens were tested under constant heat flux conditions for certain durations. However, in real ballistic re-entries, heat flux experienced by spacecraft varies with time. The spacecraft’s subjected heat flux is zero before the point of re-entry, then it starts to increase after the point of re-entry. It peaks within the atmosphere, and subsequently, it decreases and ultimately reaches zero before landing on the ground [36]. Hence, it becomes important to test the candidate materials for spacecraft heat shield applications at transient time-varying heat flux test conditions to obtain more accurate material responses. 

Moreover, studies that have included time-varying heat flux test conditions are rare; one example that could be found is in [37], in which the peak heat flux was 2.02 MW/m^2^, but here in this study, the peak value of the transient heat flux test condition is 9.4 MW/m^2^. 

One parameter that most studies have not commonly addressed is the material’s internal temperature response during the cool-off period, i.e., after the material’s exposure to heat flux test conditions has ended. As seen in our previous study [16], the material’s internal temperatures tend to increase significantly during the cool-off period compared to the values measured during the test duration. As mentioned earlier, one exception seen in our previous study was the cork material, whose internal temperatures only showed very slight elevations during the cool-off period, but that was for a lower heat flux test condition of 3.25 MW/m^2^. The main objective of the current study is to determine whether it is possible to maintain the internal temperatures of the specimens below the design limit of 453.15 K (i.e., 180 °C), even during the cool-off period.

In this study, we have addressed two conditions: (1) transient heat flux test conditions and (2) the material’s internal temperature response during the cool-off period, which are not commonly used or examined in other studies. 

## 2. Materials and Methods

### 2.1. Specimens

**Figure 1:**
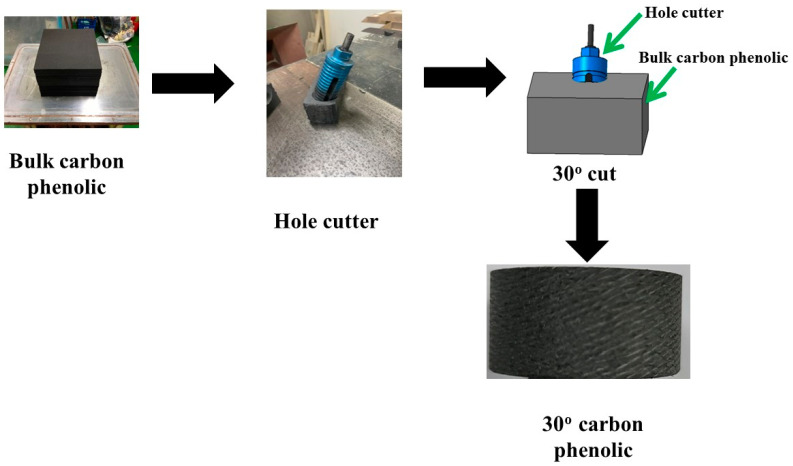
Carbon–phenolic material fabrication process.

Figure 1 shows the fabrication process of the carbon–phenolic material used in this study (final density ≈ 1340 kg/m^3^). First, a bulk carbon–phenolic block was fabricated by combining a rayon-based carbon fabric with a resol phenolic resin, followed by vacuuming and hydro-clave processes. The desired lamination angle of 30° was obtained by cutting the processed bulk carbon–phenolic block using a hole cutter at an angle of 30°. 

The fabrication process of the silica–phenolic material (final density ≈ 1791 kg/m^3^) was identical to that of the carbon–phenolic material. First, a stack of silica sheets was arranged and impregnated with the resol phenolic resin, followed by vacuuming and hydro-clave processes. Finally, the processed bulk silica–phenolic block was cut using the hole cutter at an angle of 30° to obtain the lamination angle of 30°.

The cork material used in this study was Cork P50, with an approximate density of 497 kg/m^3^. Recently, Cork P50 was used as heat shield material for the von Karman Institute for Fluid Dynamics (VKI)’s QARMAN program [38].

**Figure 2:**
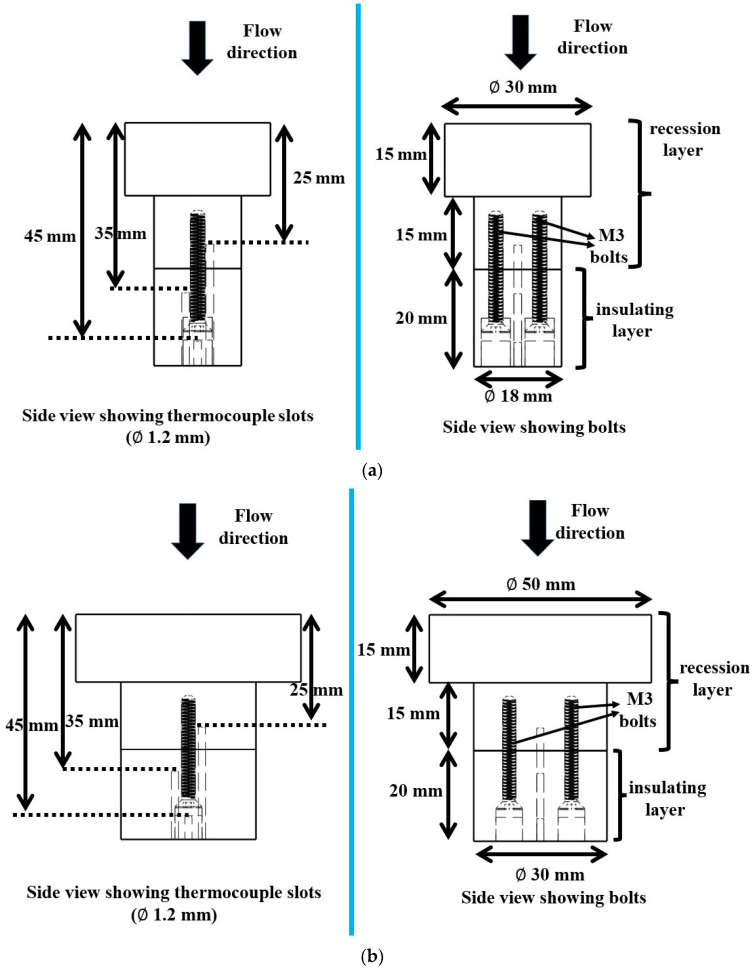
(**a**) Specimen dimensions (smaller size). (**b**) Specimen dimensions (larger size).

Figure 2a,b show the specimen dimensions. The actual specimen photographs are shown in Figures 10 and 15. Each of the smaller size specimens had a stagnation surface diameter, i.e., the surface that was exposed to the test flow, of 30 mm, and these specimens were re-purposed from our earlier study [16] for preliminary investigations at the stationary test condition. The objective of the stationary tests was to evaluate whether the combination of recession and insulating layers could withstand an intermediate heat flux test condition between the minimum and maximum values used in the transient test condition. Each of the larger specimens used for the transient test conditions had a stagnation surface diameter of 50 mm.

All specimens had a T-shape and a total length of 50 mm each. The thickness of each specimen’s insulating layer (either cork or silica–phenolic; for both sizes) was 20 mm. For each larger specimen, the insulating layer diameter was 30 mm, and for each smaller specimen, the insulating layer diameter was 18 mm. Each specimen’s recession layer had a lower stem section with a thickness of 15 mm and a diameter of either 30 mm or 18 mm, depending on the size. Additionally, the thickness of the recession layer’s top stagnation surface was also 15 mm.

The recession layers and insulating layers were attached to each other using commercially available steel M3 bolts that were 25 mm long, along with 3 mm thick aluminum washers. Two bolts were used for each specimen, with one aluminum washer per bolt. The bolt drill holes were sealed with corresponding insulating material after bolting the recession layers and insulating layers together.

Each specimen was machined with three slots, enabling internal temperature measurements at three locations, i.e., 25, 35, and 45 mm from the stagnation point. The thermocouples at 25 mm and 35 mm locations were used to observe temperature variation across the intersection of the recession layer (carbon–phenolic) and the insulating layer (silica–phenolic or cork), as these thermocouples were inserted 5 mm above and below the intersection of the recession layer and the insulating layer, respectively. The purpose of the thermocouple at the 45 mm location was to obtain the temperature information deep inside the insulating layer; for this reason, the thermocouple insertion point was 15 mm below the intersection of the recession layer and the insulating layer, i.e., 5 mm above the specimen bottom. Each thermocouple slot was 1.2 mm in diameter, and each specimen’s three thermocouple slots were machined in a straight line in the space between two bolts, the center slot being the 45 mm slot. This straight-line arrangement of thermocouples was adopted due to engineering constraints imposed by the usage of bolts, whereas a coaxial arrangement of thermocouples was used in our previous study [16]. The distance between each thermocouple slot’s center was 3 mm. Because of limitations imposed by the specimen holder and the presence of bolts, it was not possible to insert all thermocouples directly below the specimen stagnation point. As a result, it was only convenient to insert the 45 mm thermocouple directly below the specimen stagnation point, while the other two thermocouples were inserted at the closest possible locations on either side.

**Figure 3:**
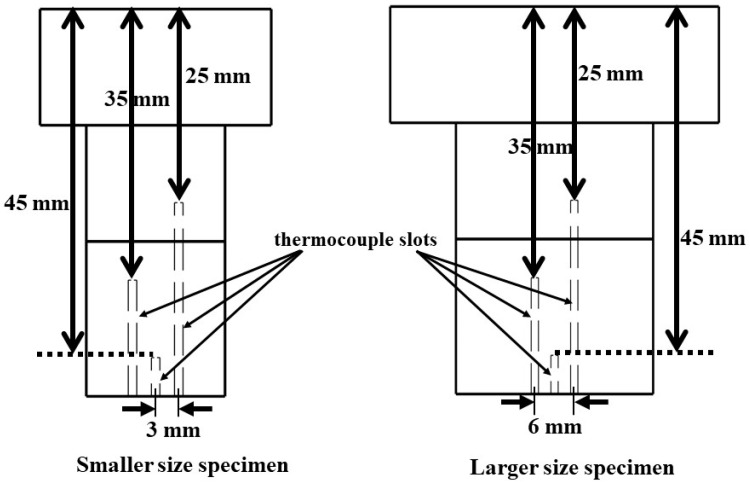
Specimen thermocouple slots (bolts and bolt insertion holes not shown).

Figure 3 only displays the thermocouple slots within the specimens, with the bolts and bolt insertion holes not shown for clarity.

**Figure 4:**
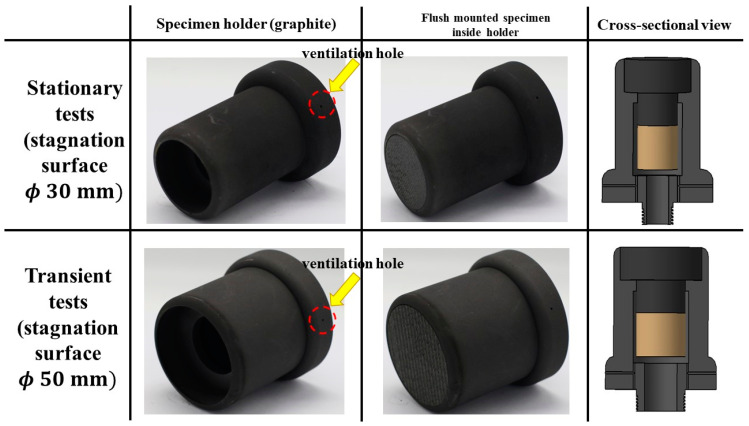
Specimen holders.

During the PWT tests, the specimens were flush mounted in specimen holders made of graphite (see Figure 4) to minimize any heat transfer from lateral directions. Each specimen holder was also equipped with two ventilation holes downstream, as air trapped inside the specimen holders tends to expand because of the high temperatures during the test.

### 2.2. Experimental Setup

The PWT used for this study is a 0.4 MW class segmented-type arc-jet plasma PWT, capable of producing supersonic plasma flows in the range of either Mach 2 or Mach 3, depending on the dimensions of the convergent-divergent flow exit nozzle used. The exit nozzle used for this study has an exit diameter of 16 mm and a throat diameter of 10.6 mm, which produces supersonic flow in the range of Mach 2 [39]. The main components of the PWT system are a gas supply manifold, segmented type arc plasma torch, vacuum test chamber, diffuser, heat exchanger, cooling water supply, DC power supply, and vacuum pump system. The vacuum test chamber contains a four-armed displacement mechanism that enables the mounting of intrusive flow diagnostic probes and specimens, allowing them to be exposed to the test flow. The vacuum test chamber’s displacement mechanism is remotely controlled and enables time-controlled motion of probes and specimens in both parallel and perpendicular directions to the test flow, as well as rotating motion. The vacuum test chamber has visual ports that enable the use of optical devices such as a pyrometer, high-speed camera, and camcorder. 

The experimental procedure in this study involved mounting the specimens on the displacement mechanism, sealing the vacuum test chamber, and supplying cooling water to the PWT components. Next, the vacuum pump system was operated to achieve the desired low test chamber pressure. After reaching the desired pressure in the test chamber, a stream of argon gas was injected into the segmented arc plasma torch using the gas supply manifold. The argon stream was directed toward the test chamber due to the high injection pressure and low chamber pressure. Next, DC power was applied between the plasma torch’s dual pairs of electrodes, and as a result, an electric arc was struck between the electrode pairs through the argon stream. Next, a mixture of air and argon, i.e., the working gas, was injected into the plasma torch through its constricted segmented packs. Due to the high thermal exchange between the electric arc and the working gas, nitrogen and oxygen molecules underwent dissociation and ionization, resulting in the generation of air plasma. The extrusion of the generated air plasma through the convergent–divergent exit nozzle into the test chamber resulted in a supersonic air plasma flow to which the specimens were exposed. Additional details on the PWT specifications and schematics can be found in [39,40]. Table 1 shows the PWT operating conditions used for this study.

The stagnation point cold wall heat flux in the axial direction of the PWT test flow was measured using a water-cooled Gardon gauge prior to the specimen tests. 

Each stationary test was conducted at 7.5 MW/m^2^ for 50 s, with the corresponding distance from the torch exit nozzle being 170 mm. During the transient tests, each specimen was inserted into the test flow at a distance of 180 mm away from the nozzle exit and then moved to a distance of 120 mm away from the nozzle exit while being exposed to the flow for 50 s. Once it reached the 120 mm distance, there was an approximately 10 s delay to reset the displacement mechanism before reversing the motion back to the 180 mm distance for another 50 s. Therefore, in the transient tests, each specimen’s total exposure time to the test flow was approximately 110 s. The heat flux value measured by the Gardon gauge at the 120 mm distance is 9.4 MW/m^2^. At the 180 mm distance, the heat flux was determined empirically as 6.25 MW/m^2^ using previously obtained experimental values. The reason for the empirical estimation was the unavailability of the Gardon gauge due to minor damages sustained during the previous measurement campaign.

Thus, the high-peak portion of an interplanetary spacecraft’s re-entry heat flux trajectory was simulated in the PWT using the transient test condition (6.25 MW/m^2^ → 9.4 MW/m^2^ → 6.25 MW/m^2^). Table 2 summarizes the specimen test conditions.

During the stationary tests, a two-color pyrometer (IMPAC series ISQ 5 MB 14 model from LumaSense Technologies, Sanat Clara, CA, USA, with a measurement range from 1273.15 K to 3273.15 K) was used to measure the specimen stagnation point temperatures. The pyrometer measurement was not possible for the transient tests due to the movement of the specimens. Two camcorders (specimen front view and side view) and a high-speed camera were used to observe and record specimen reactions. During the tests, specimen internal temperatures were measured using K-type thermocouples. The National Instruments NI cDAQ-9178 (a compact data acquisition USB chassis) with a thermocouple input module NI 9212 was used as the thermocouple data acquisition system. Three-dimensional surface measurements of each specimen’s exposed surface (i.e., the stagnation surface) were taken before and after the tests using a 3-D optical/non-contact VR-5200 measurement system from Keyence, Osaka, Japan. The purpose of these measurements was to study the morphological changes caused by ablation. After testing, magnified images of a selected specimen’s recession layer (both surface and cross-sectional) at the stagnation point were obtained using a Smartzoom 5 digital microscope from ZEISS, Oberkochen, Germany. The images were then compared with the before-test images obtained from an untested specimen.

**Figure 5:**
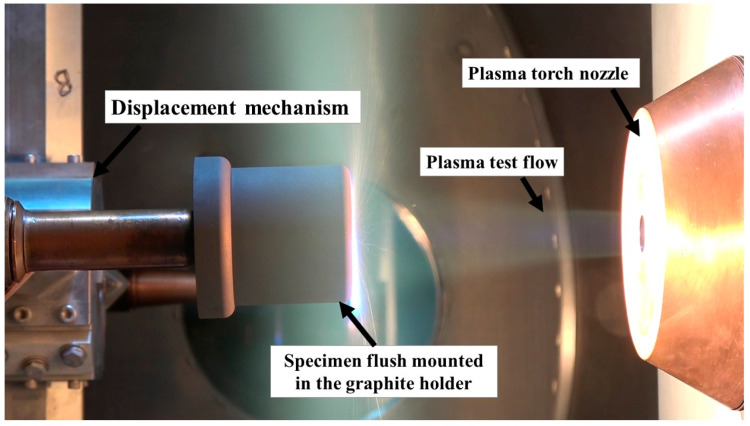
Photograph of a specimen test.

Figure 5 shows a photograph of a specimen mounted on the displacement mechanism inside the vacuum test chamber and exposed to the plasma test flow.

## 3. Results and Discussion

### 3.1. Stationary Tests

Two smaller-size specimens, namely SP-30-1 (with silica–phenolic insulating layer) and cork-30-1 (with cork insulating layer), were subjected to the stationary test condition. As mentioned earlier, the purpose of the stationary tests was to confirm whether these two specimens could withstand the test condition of 7.5 MW/m^2^ for 50 s. This value was chosen as it is closer to the average of the maximum and minimum heat flux values of the transient test condition. One of the main focuses of the stationary tests was to check the stability of the mechanical bonding between the outer recession layer (i.e., the 30° carbon–phenolic layer) and the inner insulating layer (i.e., either the silica–phenolic or cork layer) of the specimens. 

**Figure 6:**
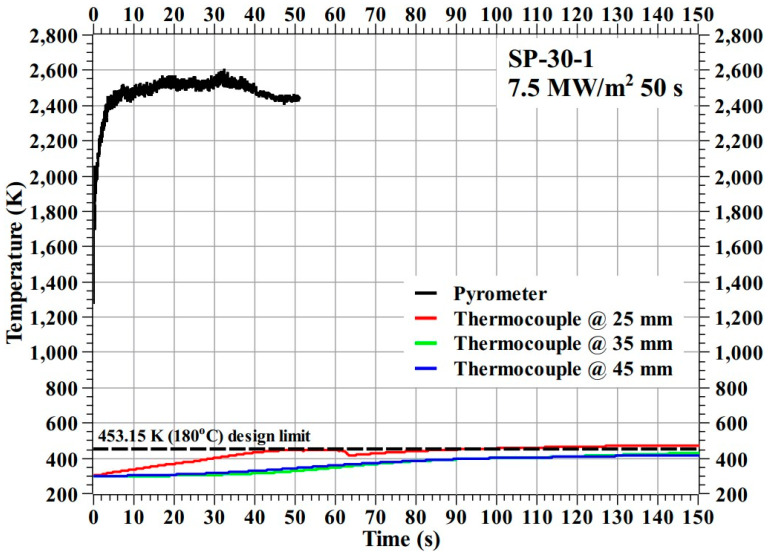
SP-30-1 specimen temperature response.

Figure 6 shows the temperature response of the SP-30-1 specimen during the test.

Figure 6 shows that the SP-30-1 specimen’s stagnation point temperature reached a maximum value of 2610.85 K during the test. The internal temperatures of the SP-30-1 specimen at the 35 mm and 45 mm locations were below the design limit of 453.15 K (i.e., 180 °C), whereas the internal temperature at the 25 mm location increased very slightly above the design limit, by approximately 20 K, after the end of the exposure time, i.e., during the cool-off period. The trends shown by the internal temperatures of the SP-30-1 specimen during the cool-off period are nearly flat, which is contrary to the trends shown by the carbon–phenolic specimens in our previous study [16], where internal temperatures tended to rise during the cool-off periods. This represents a significant improvement. Additionally, the SP-30-1 specimen did not exhibit any adverse material reactions during the test, indicating that its ablation process was normal. After-test inspection of the SP-30-1 specimen showed that the mechanical bonding between the specimen’s recession layer and the inner insulating layer of silica–phenolic was intact; there were no separations or gaps formed due to the test. Overall, the SP-30-1 specimen results were satisfactory. 

**Figure 7:**
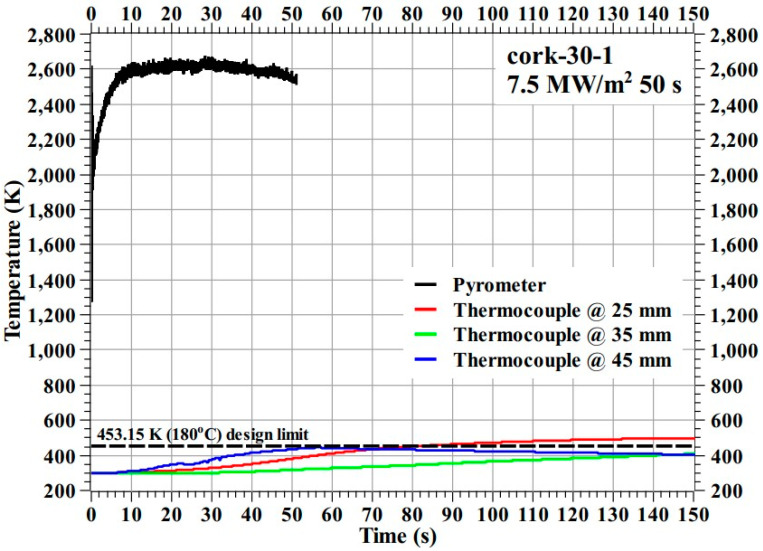
Cork-30-1 specimen temperature response.

Figure 7 shows the temperature response of the cork-30-1 specimen during the test.

Figure 7 shows that the cork-30-1 specimen’s overall temperature response was almost identical to that of the SP-30-1 specimen. During the test, the stagnation point temperature of the cork-30-1 specimen reached a maximum value of 2675.75 K. During the cool-off period, the internal temperature of the cork-30-1 specimen at the 25 mm location increased very slightly above the design limit value of 453.15 K (i.e., 180 °C) by approximately 46 K, while the internal temperatures at other locations were below the design limit. After the test, the mechanical bonding between the layers of the cork-30-1 specimen remained intact. During the cool-off period after the cork-30-1 specimen’s exposure to the plasma test flow, smoke was observed emanating from the specimen. This observation was later confirmed by analyzing video recordings of the test. In contrast, this phenomenon was not observed in the SP-30-1 specimen. As this smoke was emitted after the end of the ablation process, i.e., after the specimen’s exposure to the plasma test flow, it should not be confused with the pyrolysis gas which is produced during the ablation process. The emitted smoke could be due to the evaporation of water content in the cork layer, which can cause shrinkage of the cork layer’s volume. This may seriously affect the spacecraft’s structural integrity during atmospheric re-entry. This observed phenomenon requires detailed investigation; therefore, it was decided not to subject the carbon–phenolic–cork ablator material (i.e., the material with 30° carbon–phenolic as the outer recession and Cork P50 as the inner insulating layer) to transient tests until the exact cause and repercussions of the observed phenomenon are fully understood. 

**Figure 8:**
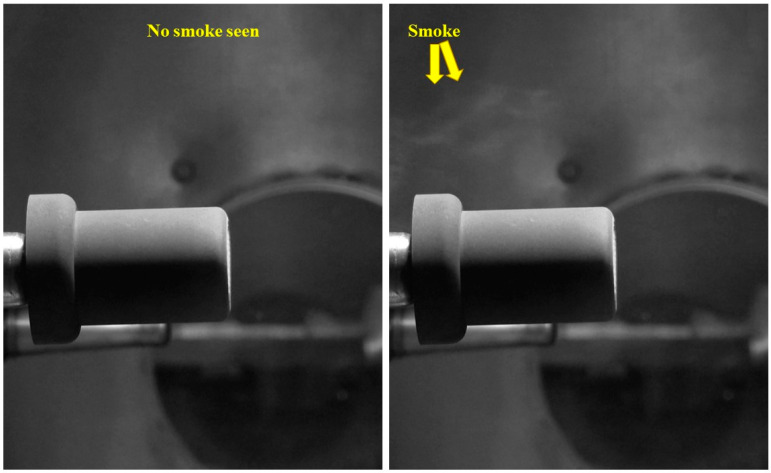
Cork-30-1 specimen cool-off period smoke emission.

Figure 8 shows two consecutive frames from a camcorder recording of the cork-30-1 specimen during the cool-off period, with smoke visible in one of the frames.

**Figure 9:**
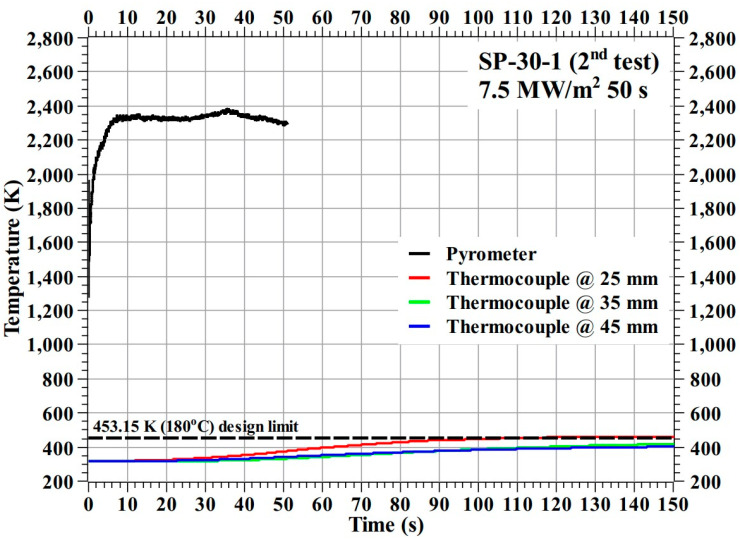
SP-30-1 specimen temperature response from the 2nd stationary test.

To confirm twice that the mechanical bonding in the carbon–phenolic–silica–phenolic ablator could withstand the longer test duration of approximately 110 s in the transient test, the SP-30-1 specimen was again subjected to the stationary test, i.e., at 7.5 MW/m^2^ for another 50 s. After-test verification confirmed the SP-30-1 specimen’s recession layer and insulating layer were still securely bonded together without any separations or gaps in between. Additionally, the SP-30-1 specimen’s surface ablation reaction observed during the test was normal. Figure 9 shows the temperature response of the SP-30-1 specimen during the 2nd stationary test. The internal temperatures of the SP-30-1 specimen during the 2nd test were almost similar to those during the 1st test. A comparison of the SP-30-1 specimen’s stagnation point temperatures shows that the maximum stagnation point temperature reached during the 2nd test was 221.70 K lower than that measured during the 1st test. The difference in stagnation point temperature is due to changes in the SP-30-1 specimen’s surface emissivity and catalycity, resulting from the alterations in surface chemistry and morphology caused by the 1st stationary test.

**Figure 10:**
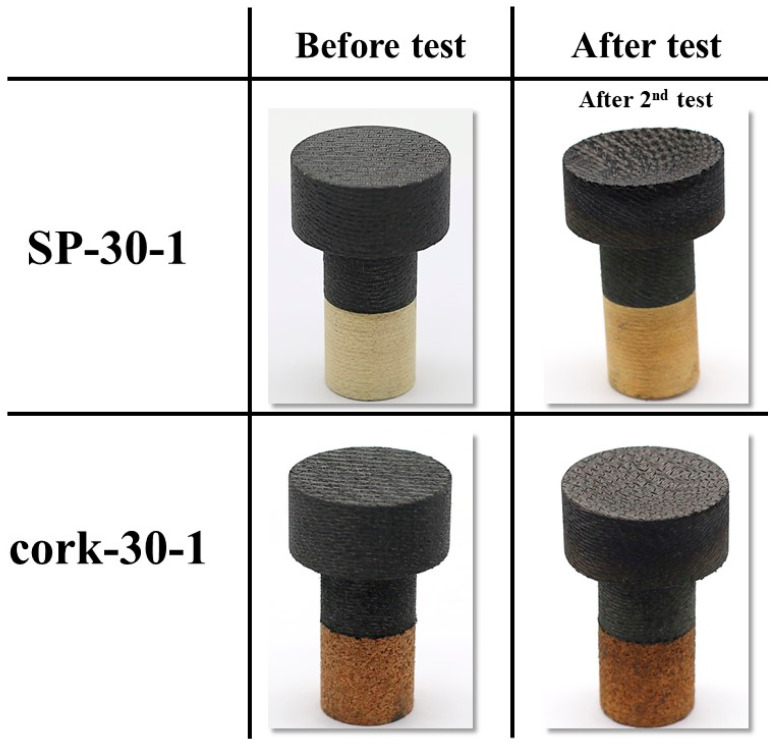
SP-30-1 (7.5 MW/m^2^, 50 s, 2 times) and cork-30-1 (7.5 MW/m^2^, 50 s) before and after test images.

**Figure 11:**
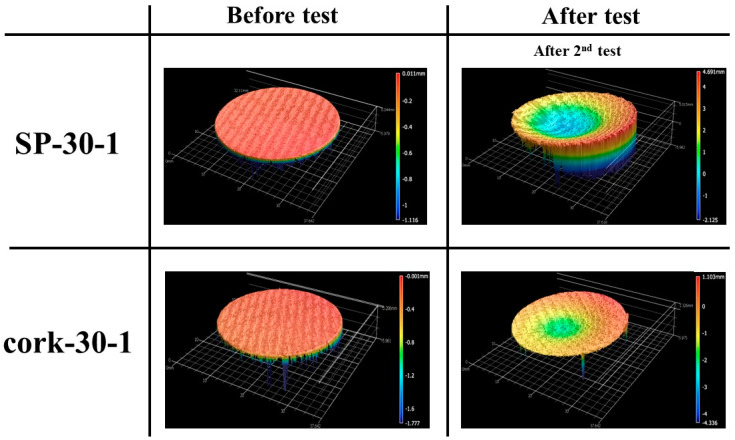
SP-30-1 (7.5 MW/m^2^, 50 s, 2 times) and cork-30-1 (7.5 MW/m^2^, 50 s) exposed surface morphology changes.

Figure 10 shows the before and after test images of the specimens SP-30-1 and cork-30-1. After-test photographs in Figure 10 show that, apart from slight darkening, the insulating layers were not affected by the tests, and the ablation process was confined to the recession layers, as intended. Figure 11 shows the before and after tests exposed surface morphologies of the specimens SP-30-1 and cork-30-1, taken using the three-dimensional optical/non-contact measurement system (i.e., the VR-5200 measurement system). It is important to note that the after-test images of the SP-30-1 specimen shown in Figure 10 and Figure 11 were taken/measured after its second stationary test. Figure 10 and Figure 11 show that, as expected, recession in the SP-30-1 specimen was more severe than that seen in cork-30-1 due to twice the test duration. This can be confirmed by the recession values, i.e., the difference between the length of the specimen before and after the test, which are shown in Table 3. The recession value of the SP-30-1 specimen is approximately 2-fold greater than that of the cork-30-1 specimen. Table 3 also shows that the SP-30-1 specimen’s mass loss was 1.6-fold greater than that of the cork-30-1 specimen.

### 3.2. Transient Tests

**Figure 12:**
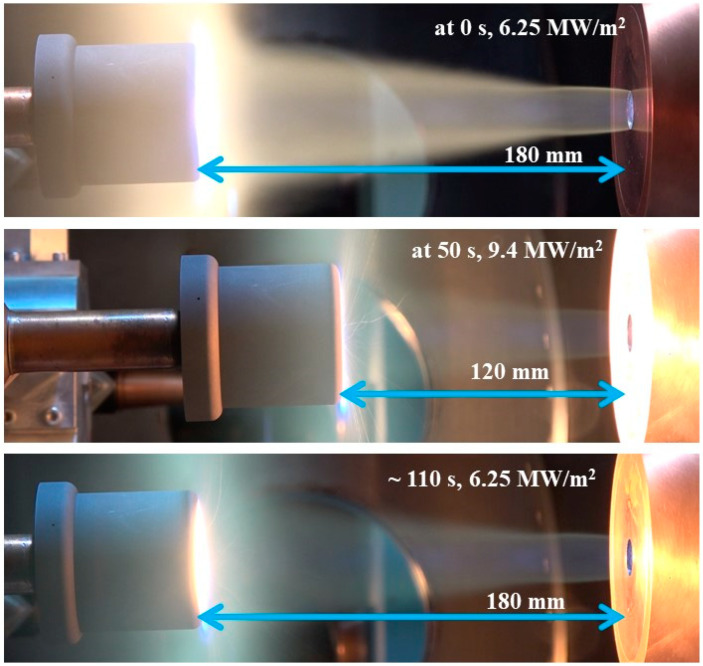
Specimen transient test.

Two larger-size specimens, namely SP-50-1 and SP-50-2, i.e., the specimens with a silica–phenolic insulating layer, were subjected to the transient test condition. Figure 12 displays a compilation of pictures that were taken during a transient test, depicting the motion of the specimens in the plasma test flow.

**Figure 13:**
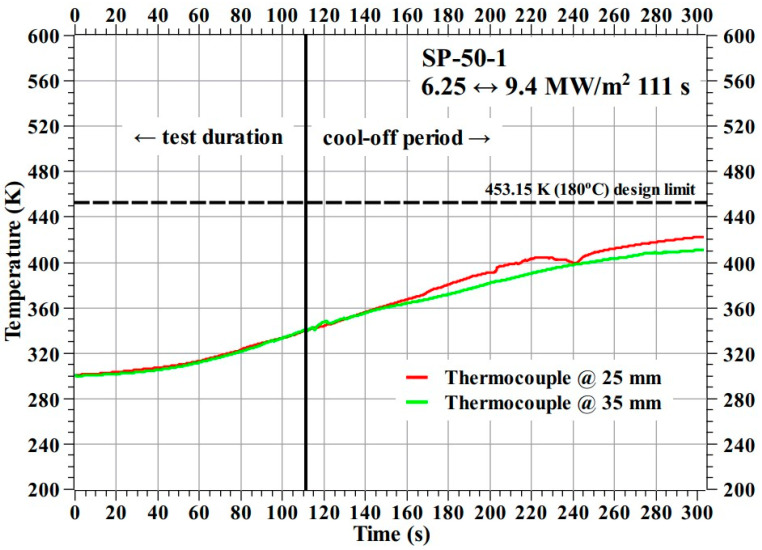
SP-50-1 specimen temperature response.

Figure 13 shows the temperature response of the SP-50-1 specimen during the test. During the SP-50-1 specimen test, the thermocouple at the 45 mm location, i.e., the farthest location from the specimen’s stagnation point, failed for unknown reasons. The SP-50-1 specimen’s temperature response during both the plasma test flow and the cool-off period clearly indicated that the internal temperatures at the 25 and 35 mm locations remained well below the design limit of 453.15 K (i.e., 180 °C), as required.

**Figure 14:**
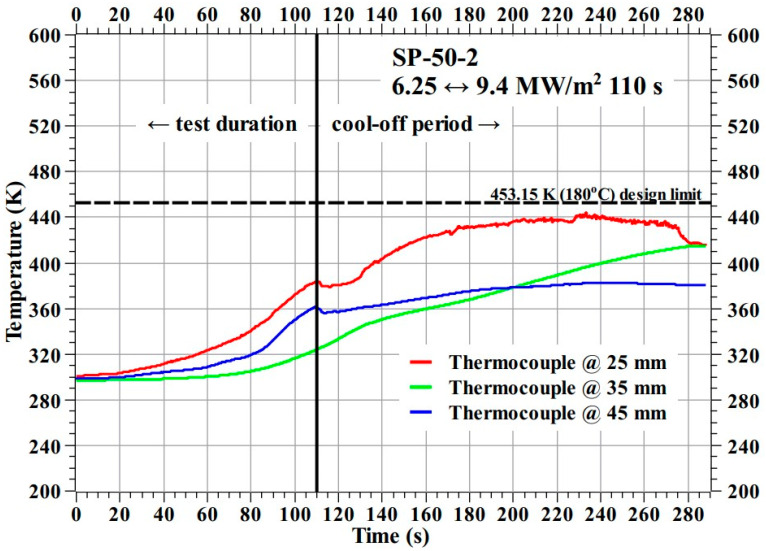
SP-50-2 specimen temperature response.

Figure 14 shows the temperature response of the SP-50-2 specimen during the test. Here, there were no thermocouple failures, and a better-defined temperature response was obtained compared to that of the SP-50-1 specimen. The SP-50-2 specimen’s temperature data confirm that the temperature response seen in the SP-50-1 specimen, and the temperature data from both transient tests confirm that the developed ablator (i.e., carbon–phenolic–silica–phenolic ablator) can maintain its internal temperatures well below the design limit of 453.15 K (i.e., 180 °C), thus satisfying the main objective of this study.

**Figure 15:**
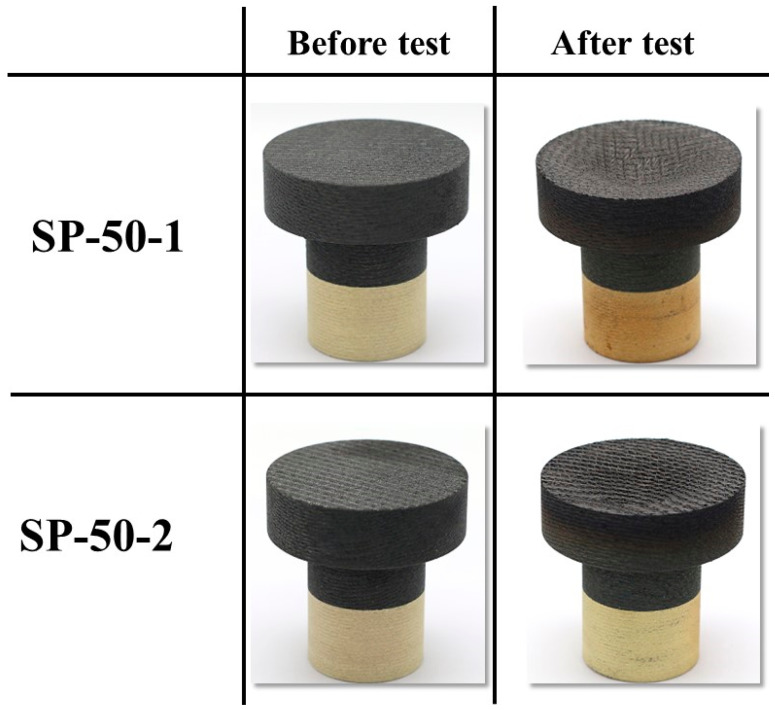
SP-50-1 (6.25↔7.5 MW/m^2^, 111 s) and SP-50-2 (6.25↔ 7.5 MW/m^2^, 110 s) before and after test images.

**Figure 16:**
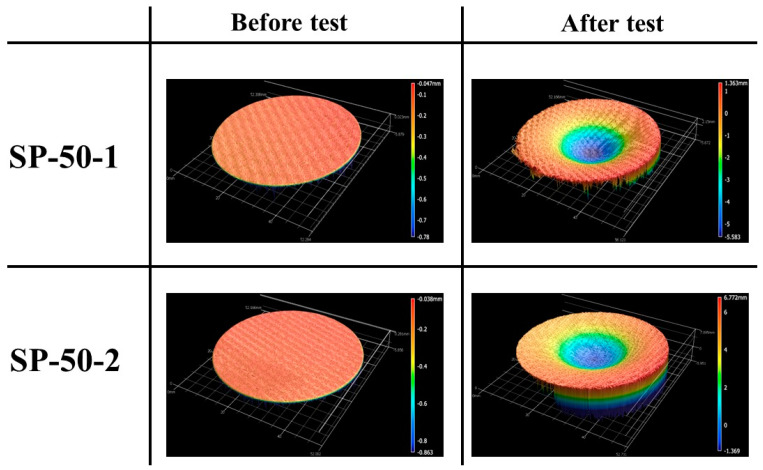
SP-50-1 (6.25↔7.5 MW/m^2^, 111 s) and SP-50-2 (6.25↔ 7.5 MW/m^2^, 110 s) exposed surface morphology changes.

Figure 15 shows the before and after test images of the specimens SP-50-1 and SP-50-2. As expected, the mechanical bonding between each specimen’s outer recession carbon–phenolic layer and insulating silica–phenolic layer was intact without any gaps or separations. Figure 16 shows the before and after tests exposed surface morphologies of the specimens SP-50-1 and SP-50-2. Figure 15 and Figure 16 show that the recessions caused by ablation in specimens SP-50-1 and SP-50-2 were identical, as confirmed by the similar recession values shown in Table 4. Table 4 also indicates that the mass losses were similar for both specimens. Compared with the specimen SP-30-1, which was tested twice at stationary test conditions, the transient test specimens SP-50-1 and SP-50-2 showed approximately 2-fold and 1.4-fold greater average mass loss and recession values, respectively. This indicates that the transient test condition was nearly two times more severe than the stationary test condition tested twice.

In Figure 6, Figure 7, Figure 9, Figure 13 and Figure 14, it can be seen that some location-wise temperature values are nearly identical to other location temperature values, especially the temperatures at 35 and 45 mm in the stationary tests. This is attributed to the presence of metallic bolts inside the specimens. These steel bolts are good thermal conductors and act as heat conduits that equalize the heat in the measurement locations.

**Figure 17:**
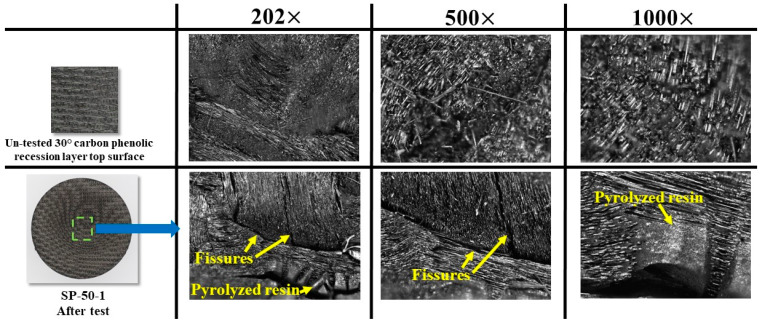
SP-50-1 specimen exposed surface magnified images comparison.

Figure 17 shows a comparison of magnified images at 202×, 500×, and 1000× magnification levels for an untested carbon–phenolic recession layer’s top surface and the SP-50-1 specimen’s stagnation point after the test. The magnified images are useful for understanding the specimen ablation process. Figure 17 clearly shows that well-developed fissures were formed on the SP-50-1 specimen’s surface during the test. These fissures were formed and developed due to the percolation of pyrolysis gas to the specimen’s surface during the test. Leftover pyrolyzed resin in the form of clumps was also formed on the SP-50-1 specimen’s surface during the test, as seen in Figure 17. 

**Figure 18:**
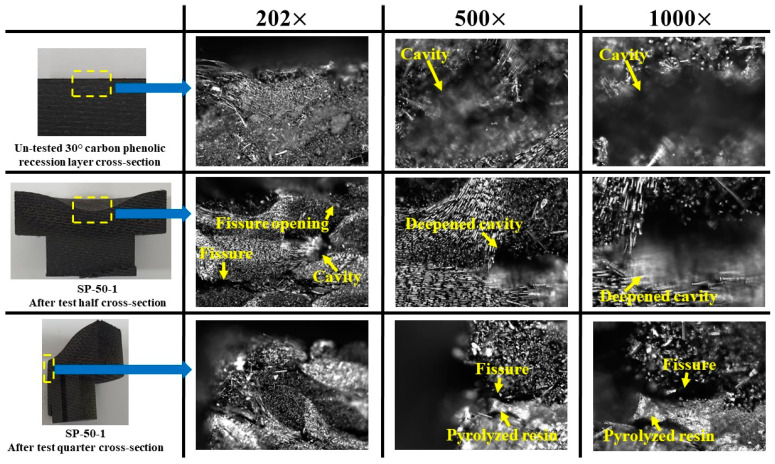
SP-50-1 specimen cross-section magnified images comparison.

Figure 18 shows a comparison of magnified images at 202×, 500×, and 1000× magnification levels for cross-sections of an untested carbon–phenolic recession layer and the SP-50-1 specimen after the test. After separating the carbon–phenolic layer and silica–phenolic layers, two different cross-sections were made for the SP-50-1 specimen: a half cross-section, which means that the specimen was cut symmetrically into two at the stagnation point, and a quarter cross-section, which means that one half of the specimen was further cut symmetrically into two at the stagnation point, quartering the specimen. The SP-50-1 specimen’s cross-sections clearly showed that the material recession due to the ablation was restricted only to the carbon–phenolic layer. At the 202× magnification level, the internal structural changes caused by the test are clearly visible. Undersurface cavities seen at the 500× and 1000× magnification levels of the untested carbon–phenolic layer are shallow, but these cavities appear to be deepened by the action of percolating pyrolysis gas in the SP-50-1 specimen. Cross-section images of the SP-50-1 specimen clearly show that fissures were also formed beneath the surface. In the half cross-section image at the 200× magnification level, one can see an internal fissure connected to a surface opening via an internal cavity. Pyrolyzed resin residue was also found to be clumped inside these internal fissures, as seen in the quarter cross-section images of the SP-50-1 specimen at the 500× and 1000× magnification levels. Based on observations in Figure 17 and Figure 18, the specimen’s ablation mechanism can be explained as follows: during the test, pyrolysis gas is generated and percolates to the specimen’s exposed surface, i.e., the stagnation surface, creating internal fissures and deepening existing cavities. Additionally, the carbon sublimation process causes the remaining resin to separate, which clumps together on the surface and in the gaps formed by the fissures.

### 3.3. Silica–Phenolic Thermal Conductivity

**Figure 19:**
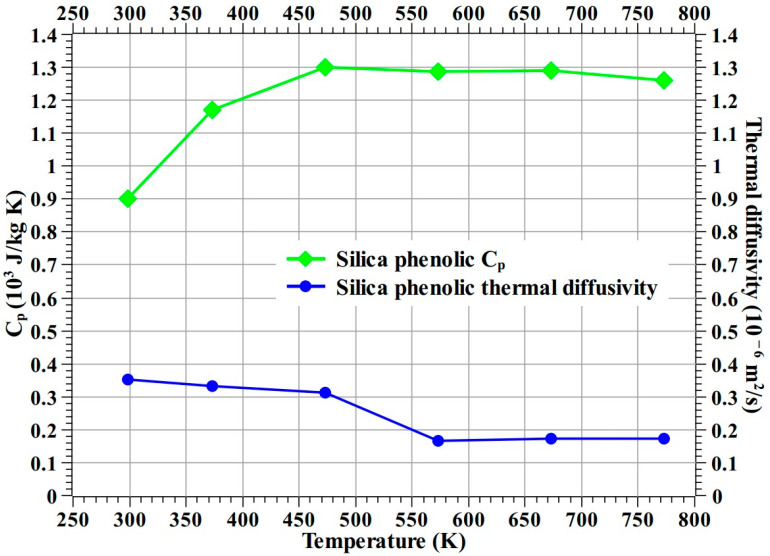
C_p_ and thermal diffusivity of silica–phenolic up to 773.15 K (500 °C).

First, *C_p_* (specific heat at constant pressure) and thermal diffusivity (α) of the silica–phenolic material used in this study were measured up to 773.15 K (i.e., 500 °C) using an LFA 467 Hyper Flash apparatus from NETZSCH, Selb, Bavaria, Germany. Figure 19 shows the measured *C_p_* and thermal diffusivity values of the silica–phenolic material. Then, using the measured *C_p_* and thermal diffusivity values, the thermal conductivity (*κ*) of the silica–phenolic was determined using Equation (1) (where ρ is material density). The values for *C_p_*, thermal diffusivity, and thermal conductivity of the 30° carbon–phenolic material, i.e., the recession layer used in this study, up to 773.15 K (i.e., 500 °C) are provided in our previous study [16]. The values for thermal conductivity of the Cork P50 material up to 523.15 K (i.e., 250 °C) can be found in [38].

(1) $$\kappa =\rho \times \alpha \times C_{p}$$

**Figure 20:**
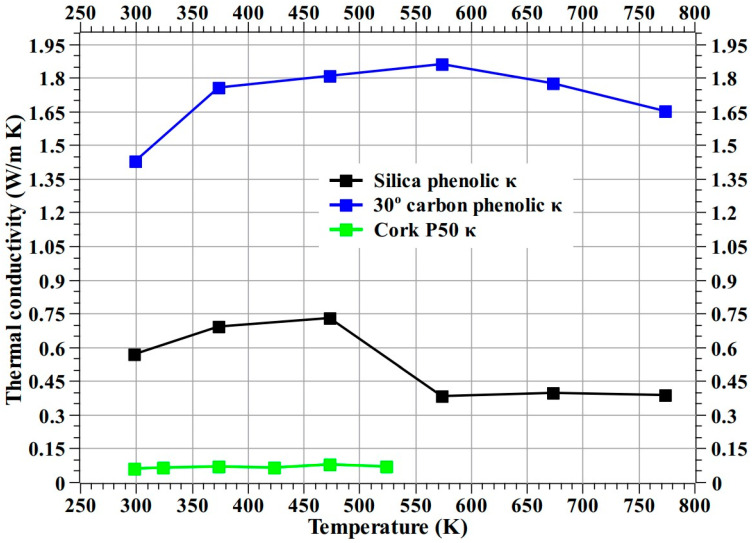
Calculated thermal conductivity of silica–phenolic along with thermal conductivities of 30° carbon–phenolic and Cork P50 from [16,38], respectively.

Figure 20 shows the calculated thermal conductivity of silica–phenolic along with thermal conductivities of 30° carbon–phenolic and Cork P50 from [16,38], respectively. According to the values presented in Figure 20, the thermal conductivity values of the 30° carbon–phenolic material are, on average, 3.5-fold greater than those of the silica–phenolic material, while the thermal conductivity values of the 30° carbon–phenolic material are on average 23.63-fold greater than those of Cork P50. The thermophysical properties of the silica–phenolic and 30° carbon–phenolic materials obtained in this study and in our previous study [16], respectively, will be useful for future analyses and aid potential improvements. 

### 3.4. Limitations and Future Plans

Due to some operational restrictions, it was not possible to simulate a complete re-entry heat flux trajectory profile in the PWT. Therefore, the specimen reactions were only examined under conditions that were close to the peak of the heat flux trajectory. The operational issues of the PWT are currently being addressed, and future work will include simulations of more complete re-entry heat flux trajectories.

Future experiments will incorporate specimens with varying thicknesses of the recession layer and the insulating layer to develop ablators with an optimal ratio between the recession layer and insulating layer.

## 4. Conclusions

Two dual-layer ablators have been developed for future Korean spacecraft applications. These ablators consist of a carbon–phenolic material as the outer recession layer and either Cork P50 or silica–phenolic as the inner insulating layer. To test the ablator specimens, a 0.4 MW supersonic arc-jet plasma wind tunnel was used with two different test conditions. A stationary test condition was used for preliminary screening, followed by a transient test condition that simulated a spacecraft re-entry heat flux trajectory. Based on visual observation made during the stationary test, it is decided that the carbon–phenolic–cork ablator needs further investigation to better understand its reaction to the test flow, and therefore, only the carbon–phenolic–silica–phenolic ablator was subjected to transient tests. 

The transient tests showed that the carbon–phenolic–silica–phenolic ablator specimens can maintain their internal temperatures at the measured locations below the targeted design limit of 453.15 K (i.e., 180 °C), not only when exposed to the test flow but also during the cool-off period. This fulfilled the main objective of this study. The reactions of the carbon–phenolic–silica–phenolic ablator specimens to the transient test were normal, and there was no damage to the internal mechanical bonding between each specimen’s recession layer and the insulating layer, thereby demonstrating excellent structural resilience.

The *C_p_*, thermal diffusivity, and thermal conductivity of the silica–phenolic material used in this study were measured for future works.

## Figures and Tables

**Table 1:** PWT operating conditions.

Operating Condition	Value
Working gas flow rate	4.14 g/s
Air percentage in working gas	95.05%
Argon percentage in working gas	4.95%
Applied total current	140 A
Applied total voltage	578.25 V
Operated total torch power	80.96 kW

**Table 2:** Specimen test conditions.

Test Condition	Heat Flux (MW/m^2^)	Distance From the Torch Exit Nozzle (mm)	Duration (s)
Stationary	7.5	170	50
Transient *	from 6.25 ^±^ to 9.4	180 to 120	50
	9.4	120	~10 ^€^
	from 9.4 to 6.25 ^±^	120 to 180	50

* Continuous exposure to the test flow, a total of ~110 s. ^±^ Empirically estimated from experimental values measured using the Gardon gauge. ^€^ Displacement mechanism direction reset time.

**Table 3:** Stationary test mass loss and recession.

Specimen	Test Condition	Mass Loss (g)	Recession (mm)
SP-30-1	7.5 MW/m^2^, 50 s (2 times)	5.72	4.63
cork-30-1	7.5 MW/m^2^, 50 s	3.59	2.47

Mass loss = specimen before test mass−specimen after test mass. Recession = specimen before test length−specimen after test length.

**Table 4:** Transient test mass loss and recession.

Specimen	Test Condition	Mass Loss (g)	Recession (mm)
SP-50-1	6.25↔7.5 MW/m^2^, 111 s	11.96	6.38
SP-50-2	6.25↔7.5 MW/m^2^, 110 s	11.56	6.89

Mass loss = specimen before test mass−specimen after test mass. Recession = specimen before test length−specimen after test length.

## Data Availability

The data will be made available on request from the corresponding author.

## Nomenclature

Abbreviations	
CPBAM	carbon–phenolic-based ablative material
ESA	European Space Agency
HVOF	high-velocity oxygen fuel
JBNU	Jeonbuk National University
PWT	plasma wind tunnel
SP	silica–phenolic
TPS	thermal protection system
Roman letter	
*C_p_*	specific heat at constant pressure (J/kg K)
Greek letters	
α	thermal diffusivity (m^2^/s)
κ	thermal conductivity (W/m K)
ρ	density (kg/m^3^)
